# Electrolyte–Electrocatalyst Interfacial Effects of Polymeric Materials for Tandem CO_2_ Capture and Conversion Elucidated Using In Situ Electrochemical AFM

**DOI:** 10.1021/acsami.4c01908

**Published:** 2024-08-01

**Authors:** Sara T. Hamilton, Maria Kelly, Wilson A. Smith, Ah-Hyung Alissa Park

**Affiliations:** † Department of Earth and Environmental Engineering, Columbia University, New York, New York 10027, United States; ‡ National Renewable Energy Laboratory, Golden, Colorado 80401, United States; § Department of Chemical and Biological Engineering and Renewable and Sustainable Energy Institute, University of Colorado Boulder, Boulder, Colorado 80309, United States; ∥ Department of Chemical and Biomolecular Engineering, University of California Los Angeles, Los Angeles, California 90024, United States

**Keywords:** Polymers, Electrified interfaces, Electrolyte, Reactive CO_2_ capture, CO_2_ capture and conversion, CO_2_ reduction

## Abstract

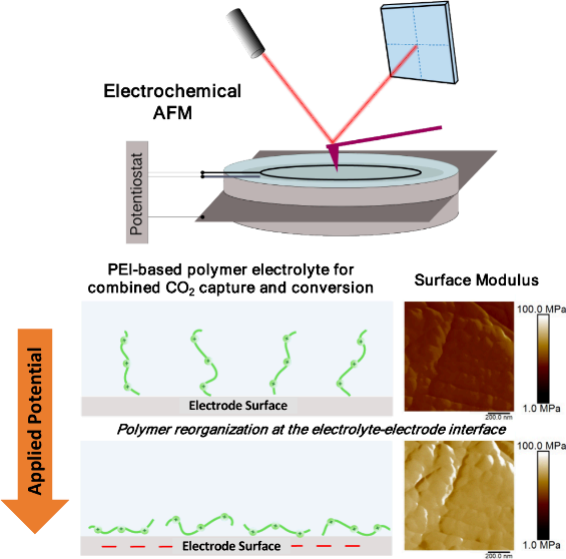

Integrating CO_2_ capture and electrochemical conversion has been proposed as a strategy to reduce the net energy required for CO_2_ regeneration in traditional CO_2_ capture and conversion schemes and can be coupled with carbon-free renewable electricity. Polyethylenimine (PEI)-based materials have been previously studied as CO_2_ capture materials and can be integrated in these reactive capture processes. PEI-based electrolytes have been found to significantly increase the CO_2_ loading, and impact selectivity and rate of product formation when compared to the conventional aqueous electrolytes. However, the influence of these materials at the catalyst–electrode interface is currently not well understood. In this study, PEI-based electrolytes were prepared and their impact on the morphology of a silver electrode performing electrochemical CO_2_ reduction (CO_2_R) was studied using in situ electrochemical atomic force microscopy (EC-AFM). The presence of PEI on the electrode surface could be distinguished based on nanomechanical properties (DMT modulus), and changes were observed as negative polarization was applied, revealing a reorganization of the PEI chains due to electrostatic interactions. These changes were impacted by the electrolyte composition, including the addition of supporting electrolyte KHCO_3_ salt, as well as CO_2_ captured by the PEI-based electrolyte, which minimized the change in surface mechanical properties and degree of PEI alignment on the electrode surface. The changes in surface mechanical properties were also dependent on the PEI polymer length, with higher molecular weight PEI showing different reconfiguration than the shorter polymer brushes. The study highlights that the choice of polymer material, the electrolyte composition, and CO_2_ captured impact the near-electrode environment, which has implications for CO_2_R, and presents EC-AFM as a new tool that can be used to probe the dynamic behavior of these interfaces during electrocatalysis.

## 1 Introduction

The increased concentration of CO_2_ in the atmosphere and its effects on global climate change has motivated extensive research on CO_2_ capture technologies from point and dilute sources, which play a key role in meeting climate targets set by the Intergovernmental Panel on Climate Change (IPCC).^1,2^ Amine scrubbing is the most mature capture technology and the current industrial benchmark for postcombustion CO_2_ capture.^2^ It has been investigated extensively over the past decade but continues to be challenged by its high parasitic energy consumption, sorbent degradation, and corrosion.^3^ These challenges have prompted research into new sorbent designs, including metal–organic frameworks (MOFs),^4,5^ ionic liquids (ILs),^6,7^ zeolites, and other porous carbon materials.^8,9^ The field of electrochemical CO_2_ reduction (CO_2_R), where captured CO_2_ is used as a feedstock and is reduced at a catalyst surface in an electrochemical cell, has also grown significantly in recent years. CO_2_R offers an attractive pathway to address the challenge of CO_2_ emissions, while simultaneously producing value-added chemicals and fuels.^10,11^

Traditionally, CO_2_ capture and electrochemical conversion have been studied independently, but there has been a recent focus on integrating the capture and conversion reactions in a “reactive capture” scheme, where the CO_2_ capture material is incorporated as an electrolyte additive, carrying the captured CO_2_ to the catalytic sites on the electrode, where CO_2_ regeneration and/or conversion is driven by an electrochemical reaction, rather than by a thermal or pressure swing.^12,13^ Coupling these two processes is advantageous from an energy efficiency and cost perspective, reducing the energy requirements for CO_2_ regeneration and removing the CO_2_ purification step.^12,14,15^ Electrochemical CO_2_ regeneration schemes offer the additional advantage that they can be coupled directly with carbon-free renewable electricity and can be implemented in a modular fashion and in a wide variety of locations. Combined CO_2_ capture and electrochemical conversion has been demonstrated in a limited number of studies using various capture agents including ionic liquids (ILs) and simple amine molecules. ILs have been extensively studied for CO_2_ capture,^16−18^ and their integration in CO_2_R schemes was found to be favorable in that they can reduce the overpotentials required for CO_2_ regeneration by bending the CO_2_ adduct.^19,20^ Several groups have reported on increased faradaic efficiencies for target products using IL electrolyte additives.^21,22^ Reactive CO_2_ capture has also been explored using amines, where carbamate is the electrochemically active CO_2_ adduct. Chen et al. reported on CO_2_ reduction in aqueous monoethanolamine (MEA) solution,^23^ and Pérez-Gallant et al. demonstrated the feasibility of integrating capture of CO_2_ by 2-amino-2-methyl-1-propanol (AMP) with conversion to formate and carbon monoxide by operating at high temperatures of 75 °C in propylene carbon solvents.^24^ Lee et al. also demonstrated the feasibility of electrochemically upgrading CO_2_ from MEA by tailoring the electrochemical double layer using alkali ions.^14^

Polymeric materials, such as nanoparticle organic hybrid materials (NOHMs), which consist of an organic polymeric canopy tethered to a nanoparticle core,^25^ are another class of CO_2_ capture materials proposed for reactive capture. Due to their favorable properties, including negligible vapor pressure and high thermal stability under oxidizing conditions,^26,27^ they have been proposed for CO_2_ capture^25,28−30^ and other electrochemical applications.^31−34^ The fundamental properties of NOHMs and their constituent polymers in electrolyte solutions have been extensively studied,^35−38^ and they were found to mediate metal electrodeposition reactions at electrode interfaces, which are of interest in various electrochemical energy storage devices.^34,39^ Because of the ability of polyethylenimine (PEI)-based NOHMs, as well as unbound PEI, to boost CO_2_ concentration in solution, their potential to act as electrolyte additives for combined CO_2_ capture and conversion has been investigated.^13,40^ Feric et al. recently demonstrated integrated capture and conversion of CO_2_ in NOHMs and PEI-based electrolytes on a silver nanoparticle catalyst and found that their addition led to a change in selectivity and reaction rate compared to conventional aqueous electrolytes.^41^

Although the impact of these polymeric CO_2_ capture materials on product distributions has been reported, because of the relative novelty of these schemes, the mechanism for the conversion reaction is still under debate. In particular, their behavior at electrified interfaces where the CO_2_ regeneration step occurs is currently not well understood. This is particularly important for CO_2_R because local near-electrode environment conditions impact electrocatalyst activity and stability, product distribution, and overpotentials for the desired conversion reaction.^42,43^ Feric et al. attributed some of the changes in selectivity observed in CO_2_R with the addition of PEI to polymer–catalyst interactions revealed by SEM-EDX. However, these particular measurements were conducted ex situ and thus provided limited information about dynamic changes occurring at the electrode while CO_2_R is occurring.^41^ In situ characterization tools such as electrochemical atomic force microscopy (EC-AFM) enable the study of electrochemical interfaces and interphases while an electrochemical potential is applied. Note that we consider EC-AFM measurements in situ rather than operando because product selectivity information is not measured concurrently. An operando EC-AFM measurement would require integration with in-line product detection, such as gas and liquid chromatography. EC-AFM can provide in situ topographical maps of sample surfaces with nanoscale resolution in a nondestructive way.^44^ When EC-AFM is performed in PeakForce tapping mode, surface mechanical properties including Young’s modulus, adhesion, and deformation from load–displacement curves can be determined.^45^ While a number of in situ EC-AFM studies have been conducted on electrochemical interfaces and interphases relevant to batteries^46,47^ and water splitting,^48,49^ the tool is relatively new in the field of CO_2_R. Nesbitt et al. recently employed EC-AFM to study CO_2_R on a gas diffusion electrode (GDE), enabling high current densities to be measured.^50^ Other recent works have used EC-AFM to observe morphology changes in metal foil or nanoparticle CO_2_R catalysts.^51,52^ No known studies to date have studied the effect of electrolyte materials for combined CO_2_ capture and conversion at the electrolyte–electrode interphase using this technique.

The goal of this work is to discern the effect of polymeric amines, a class of materials enabling combined CO_2_ capture and conversion, on the morphology of a silver electrode surface using in situ EC-AFM. Specifically, the study seeks to understand near-electrode surface effects of polyethylenimine (PEI), including whether a selective adsorption layer forms at the electrode and the effect of applied potentials relevant to CO_2_R on such a layer. PEI was chosen as the focus of this work because it is a well-characterized compound which has been extensively studied for CO_2_ capture^53−55^ and, more recently, for combined capture and electrochemical conversion by our group.^41^ The results are expected to provide insights into the behavior of the more complex NOHM-I-PEI-based system (which is based on the same chemical amine functionality, since PEI is the polymeric canopy in these materials). We prepared PEI-based electrolyte solutions and studied the topography and nanomechanical properties of Ag surfaces with PEI additives using the PeakForce QNM mode of EC-AFM. Topographical and mechanical property maps were collected as negative potentials relevant to CO_2_R were applied. We also explored the effect of electrolyte composition and CO_2_ capture by preparing and imaging electrolyte solutions with supporting electrolyte KHCO_3_ salt and under CO_2_ saturation. Finally, the effect of polymer molecular weight (MW) on morphological changes at the electrode interface were explored by preparing and imaging solutions with PEI of different MW (2000 and 25000). The findings from this work revealed that the addition of an amine-based polymer (PEI) for reactive CO_2_ capture impacts the near-electrode environment on a Ag electrode during CO_2_R, and PEI polymer chains can change conformation on the surface as the electrode is negatively polarized due to electrostatic effects, leading to changes in interfacial morphology and mechanical properties. Electrolyte composition and polymer MW were found to impact this reconfiguration phenomena and are thus important parameters to consider when elucidating interfacial morphology. This is one of the first studies of its kind to employ in situ characterization tools to study reactive capture materials at an electrified interface and therefore also serves to demonstrate the potential of this technique to provide new insights into morphology–electroactivity relationships in materials of interest for these applications. The techniques developed in this work can be extended to further understanding of polymers at charged interfaces broadly, as well as to probe other complex reactive capture media including NOHMs and ILs, which could guide their design to selectively form desired products at low overpotentials during CO_2_R.

## 2 Methods

### 2.1 Electrolyte Preparation

Binary mixtures of polyethylenimine (PEI, MW = 2000 g/mol, Polysciences Inc., and MW = 25000, Sigma) and deionized water (18 MΩ) were prepared using an analytical balance (LA-314.C, Cole Parmer) with a precision of 10^–4^ g. Salt-containing electrolytes were prepared by mixing powdered KHCO_3_ salt (Sigma-Aldrich, ACS, ≥99.0%) with the previously prepared PEI solutions to obtain electrolyte solutions with a concentration of 0.1 molal (*m*) KHCO_3_. For CO_2_-saturated solutions, the PEI-containing electrolytes were purged with CO_2_ until the saturation pH was reached (pH ∼ 7.0). For the PEI MW 2000 solutions, the concentration of the electrolyte was set to 8 wt %, which was found by Feric et al. to be the optimum PEI concentration that maximizes CO_2_ capture capacity while minimizing mass transfer limitations in the electrolyte due to viscosity effects.^41^ The polymer concentration in the case of the different MW was normalized by the number of polymer chains.

### 2.2 Electrochemical Cell Design and Assembly

**Figure 1:**
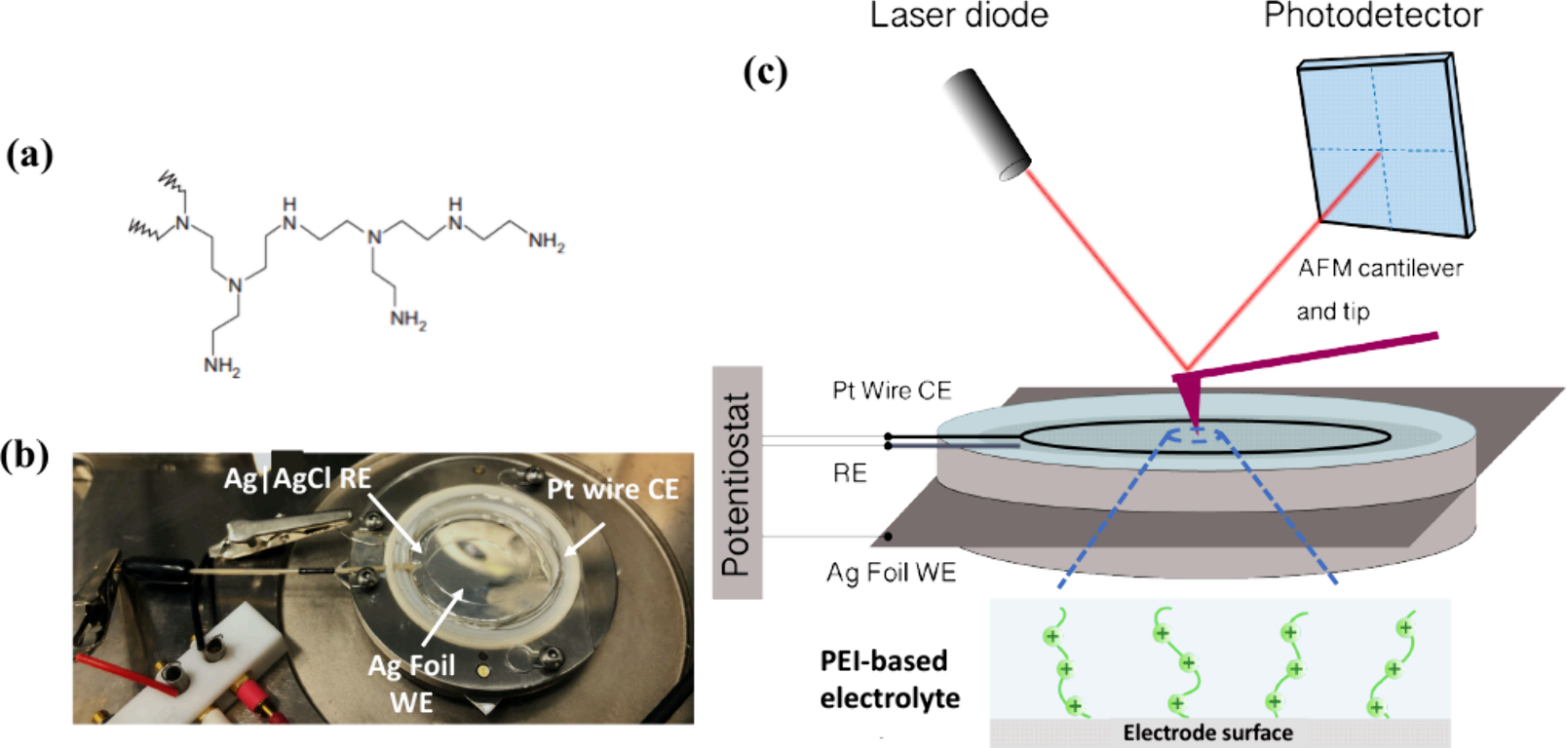
EC-AFM electrochemical cell schematic and photos used in this study. (a) Structure of branched polyethylenimine (PEI). (b) Photo of the electrochemical cell with Ag foil as the working electrode. (c) Schematic of the EC-AFM instrument used in this work.

The electrochemical cell used in these measurements was the Bruker Dimension Icon electrochemical cell for EC-AFM. A 5 × 5 cm^2^ Ag foil (99.9% metals basis, 0.127 mm thick, Thermo Scientific) was placed between the electrochemical cell stainless steel base plate and the electrolyte and was connected to a potentiostat (CH Instruments, Inc. 760E), acting as the working electrode (WE). A coiled Pt wire was used as the counter electrode (CE), and a micro-Ag|AgCl electrode (LF-1-45 coated, Innovative Instruments) was used as the reference electrode (RE). A schematic and photos are shown in Figure 1. To seal the electrochemical cell, the glass cover was slid into position and the top screws were tightened.

### 2.3 EC-AFM Measurements

A CHI 760E potentiostat was connected to the Pt wire CE, the Ag WE and the Ag|AgCl RE. Once the sample position was checked in air (*x*, *y*, *z* position on Bruker Dimension Icon stepper motors), the electrochemical cell was filled with 2 mL of the electrolyte solutions using a plastic pipette. AFM topography and nanomechanical properties images were collected simultaneously in PeakForce QNM mode using an RTESPA-525 probe (Bruker Corp., *k* = 200 N/m, *f*_0_ = 525 kHz) for the samples without polymer electrolyte and an RTESPA-150 probe (Bruker Corp., *k* = 5 N/m, *f*_0_ = 150 kHz) for the samples with polymer electrolyte. Before every experiment, the probe was calibrated for a precise measurement of its mechanical properties. The spring constant (*k*) was calculated using the Sader method. The probe radius was determined by imaging a Ti Roughness sample (RS-12M, Bruker) and analyzing the image with Bruker Nanoscope Analysis software. Nanoindentation mechanical measurements were conducted at the same time as the morphology was mapped. The imaging scan rate was set to 1 Hz (lines per second) with a resolution of 256 × 256 pixels. For samples where potentials were applied, chronopotentiometry was used to apply potentials of −0.2 V, −0.4 V, and −0.6 V vs RHE. For each of the samples measured, experiments were repeated in triplicate. Experiments were conducted at room temperature (25 °C).

### 2.4 EC-AFM Data Analysis

Nanoscope Analysis software was used for data processing. Root mean square (RMS) roughness (*R*_q_), which measures the root-mean-square deviation of a profile, was calculated using the analyze/roughness subroutine in the Nanoscope software. The mathematical expression for *R*_q_ is given in eq 1. For each of the properties reported, nanomechanical properties were averaged across 6 different spots of the 1 × 1 μm^2^ image. These were then averaged over the 3 triplicate data sets, with both average properties and standard deviations reported.
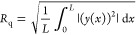
1where *L* is the length of the profile on the *x*-axis and *y*(*x*) is the variation of the height from the profile line for each data point.

## 3 Results and Discussion

### 3.1 Presence of Polyethylenimine (PEI) at Electrode Surface Discerned Based on Mechanical Properties from Peak Force QNM Mode

**Figure 2:**
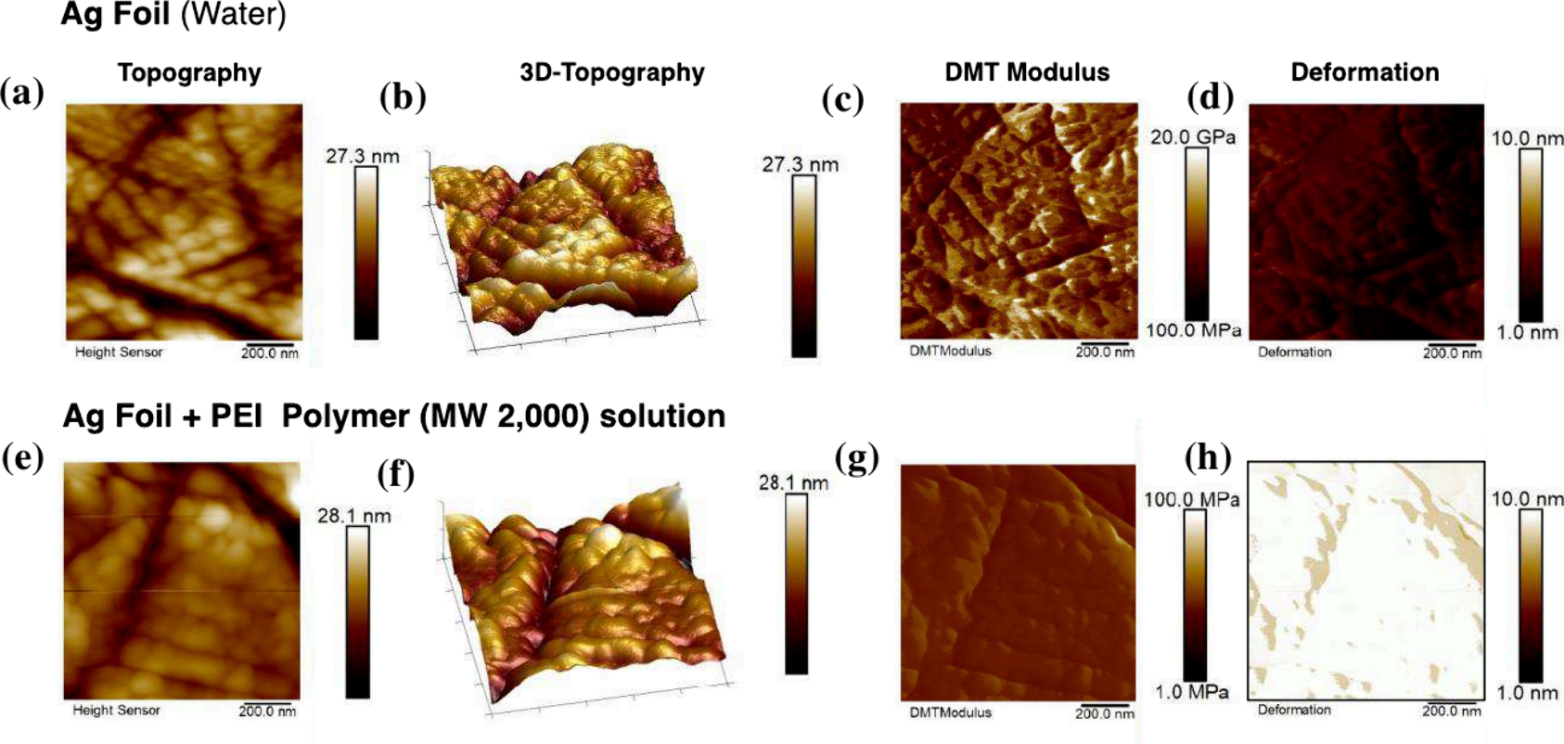
2D and 3D topography and nanomechanical property images of Ag surface without addition of 8 wt % polyethylenimine (PEI), MW 2000: (a) 2D topography, (b) 3D topography, (c) DMT modulus, (d) deformation. 2D and 3D topography and nanomechanical property images of Ag surface with addition of 8 wt % polyethylenimine (PEI), MW 2000: (e) 2D topography, (f) 3D topography, (g) DMT modulus, (h) deformation. All the images have a scan area of 1 × 1 μm^2^. Scale bars are shown at the right side of each image.

Surfaces of the Ag electrode with the addition of 8 wt % polyethylenimine (PEI) polymer MW 2000 were imaged using the EC-AFM to understand the effects of PEI on surface topography and nanomechanical properties. The electrode surface with just water as the liquid solution was also imaged to serve as a base case for comparison. Figure 2 shows the resulting images in both cases, presenting height, Derjaguin–Muller–Toporov (DMT) modulus (reduced Young’s modulus calculated according to the DMT model,^56^ see eq 2 for a full expression) and deformation within a square scan area of 1 × 1 μm^2^.
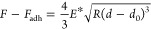
2where E* is the reduced modulus, *F* – *F*_adh_ is the force of the cantilever relative to the adhesion force, *R* is the radius of the tip, and *d* – *d*_0_ is the deformation of the sample.

Table 1 shows the average surface roughness, DMT modulus, and surface deformation of both cases. The addition of PEI to the electrolyte was found to lead to a small increase in surface roughness relative to the blank case with no PEI addition (the liquid solution is water in this case), but the changes between the two are subtle. This is likely due to the fact that the length of these PEI polymers in solution is relatively short (∼2 nm hydrodynamic diameter, as determined from dynamic light scattering^57^). However, significant differences were obvious when both surfaces were compared in terms of their nanomechanical properties. While the planar Ag surface with no additives was found to have a modulus in the range of 10s of GPa (Figure 2c), the modulus of the surface with the addition of the PEI electrolyte additive was in the MPa range (Figure 2g). The order of magnitude of these moduli values agree with reported values for planar silver foil^58^ and polymer films, respectively,^59−61^ and the large differences between the two clearly indicate the presence of a polymer layer on the Ag electrode surface. The Ag surface with reference electrolyte and PEI-containing electrolyte also differed in terms of their deformation, with the Ag surface with PEI-containing electrolyte showing higher deformation (Figure 2h), than the Ag surface with the reference sample (Figure 2d). This is expected from the DMT-modulus/deformation relation given in the SI. Since the DMT modulus is related to the material’s deformation and is a more widely reported property, for the rest of this work, the modulus is the main nanomechanical property reported and discussed. These findings suggest that there is selective PEI adsorption on the Ag electrode surface even at relatively low concentration of PEI. This is in agreement with findings from Feric et al., who reported on PEI adsorption on Ag electrode surfaces based on ex situ analysis using SEM-EDX.^41^

**Table 1:** Surface Properties of Ag Surface with Blank Electrolyte (Water) and 8 wt % PEI Electrolyte

electrolyte composition	average roughness (*R*_q_), nm	average modulus	average deformation, nm
water (base case)	4.6 ± 1.8	8.8 ± 2.1 GPa	2.8 ± 0.3
8 wt % PEI solution	5.9 ± 2.7	47.6 ± 2.1 MPa	8.6 ± 1.3

### 3.2 Changes in Structural Conformation of PEI at the Electrode Surface under Applied Potential

As discerned from differences in nanomechanical properties shown above, PEI forms an adsorbed layer on the Ag electrode even at low concentration. This finding suggests that PEI at the electrode surface would participate in CO_2_R, and the conversion of CO_2_ captured by PEI in the electrolyte would be influenced by the local structures and chemical reaction environment created by the adsorbed layer of PEI at the electrode surface. During CO_2_R, negative polarization is applied on the electrode surface. This motivated further experiments to address a question: How does the adsorbed PEI layer respond to changes in applied potential? To decouple the electrochemical reaction with the structural changes of PEI under applied potential, we first performed experiments without any supporting electrolyte or CO_2_ capture.

**Figure 3:**
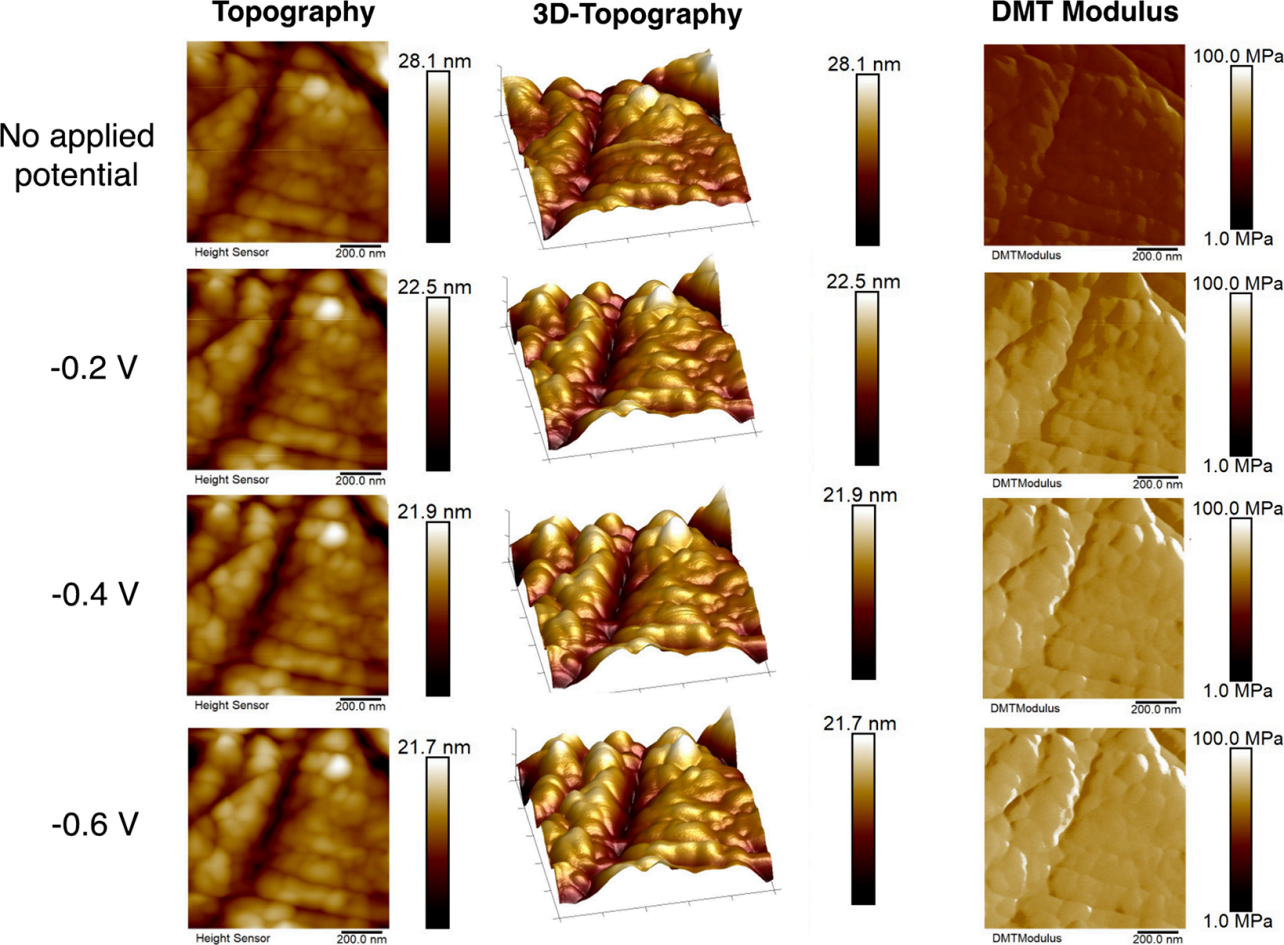
Topography and nanomechanical property mapping of Ag surface with PEI addition under no applied potential and with applied potentials in the range of −0.2 V to −0.6 V vs RHE. All of the images have a scan area of 1 × 1 μm^2^. Scale bars are shown at the right side of each image. All voltages quoted vs RHE.

Figure 3 shows the topography and nanomechanical properties of the PEI MW 2000 electrolyte with no applied potential, as well as under increasingly negative applied potentials of −0.2 V, −0.4 V, and −0.6 V vs RHE. The average values of surface roughness, DMT modulus, and surface deformation are given in Table S1. Imaging at more negative applied potentials was not possible due to distortion of the AFM probe by formation of bubbles on the surface, as has been reported by previous studies.^50^ From these AFM images, we observed an increase in the magnitude of the average DMT modulus of the surface with applied potential, suggesting that a structural rearrangement was occurring at the surface as negative polarization is applied. This can be attributed to changes in the conformation of the PEI chains in response to their interaction with the negatively charged electrode surface. Since PEI is a cationic polymer, electrostatic interactions will be a driving force for adsorption on the surface, and the polymer chains may horizontally align with the charged electrode surface when a negative potential is applied. Previous studies using spectroscopic techniques have reported on PEI adsorption at charged interfaces. Lin et al. used surface enhanced raman spectroscopy (SERS) to probe the behavior of PEI in solution at a Ag charged surface and reported that it can adsorb on these surfaces through its amino groups, with an increase of the chain order on the electrode under negative applied potentials.^62,63^ This rearrangement is consistent with the trends in nanomechanical properties observed here using EC-AFM. The change in the structural conformation of PEI at the electrode surface suggests that the selectively adsorbed polymer layer may impact the rate and selectivity of CO_2_R by altering the transport behaviors at the interface and functional groups of PEI interacting with active sites on the electrocatalyst.

### 3.3 Effect of Supporting Electrolyte and CO_2_ Capture on the Structure of PEI at the Electrode Surface under an Applied Potential Relevant to CO_2_R

Our previous study showed that ionic stimuli significantly impact the conformation of the polymeric canopy in NOHMs.^31,35^ Thus, we hypothesized that the addition of supporting electrolyte and CO_2_ capture by PEI-based electrolyte would also affect the structural conformation of PEI at the electrode interface. We therefore investigated the effect of electrolyte composition on these parameters by conducting these experiments with the addition of 0.1 *m* KHCO_3_ electrolyte salt and under CO_2_ saturation, which are the conditions under which experiments to evaluate the performance of PEI-based electrolytes for CO_2_R were previously conducted. The effect of electrolyte cations on the morphology of the electrode surface is also of broad interest because cations have been demonstrated to have an important effect on the selectivity of important electrochemical processes and impact local concentration of reaction intermediates at the electrode interface.^64,65^

**Figure 4:**
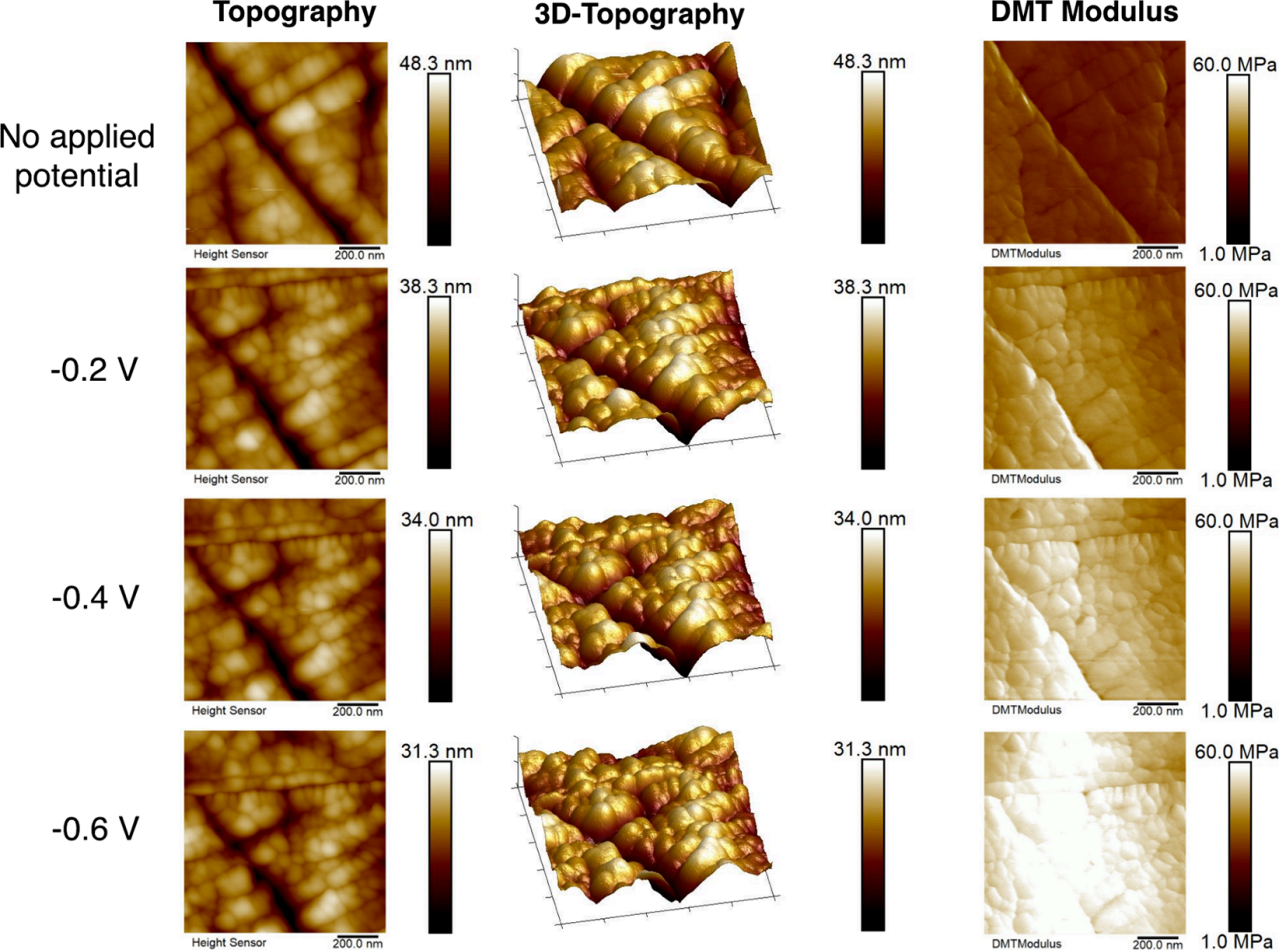
Topography and nanomechanical property mapping of Ag surface with PEI + 0.1 *m* KHCO_3_ addition under no applied potential and with applied potentials in the range of −0.2 V to −0.6 V vs RHE. All of the images have a scan area of 1 × 1 μm^2^. Scale bars are shown at the right side of each image. All voltages quoted vs RHE.

As shown in Figure 4, the addition of 0.1 *m* KHCO_3_ supporting electrolyte salt impacted the surface properties measured with the AFM. The average values of the surface roughness, DMT modulus, and surface deformation are given in Table S2. First, it can be noted that the average modulus of the PEI containing Ag surface in the case of no applied potential was lower than in the sample described in the previous section (PEI in the absence of supporting electrolyte salt). This is likely due to the salt anions screening some of the charges on the polymeric backbone, resulting in compaction of the polymeric chains due to a reduction of the intrachain charge repulsion, an effect that has been reported previously for charged polymers.^66^ Under applied potential conditions, an increase in the average modulus of the surface was still observed, but the change was not as significant as the case with no supporting electrolyte salt. These changes suggest that the supporting salt cations, which are also drawn to the double layer via electrostatic forces, impact the configuration of the polymer chains at the interface, and there is less of an alignment of the polymer chains with the electrode surface in this case. This could be due to a competition between the polymer chains and the salt cations at the electrical double layer: in the pure PEI electrolyte, the PEI chains are the only cationic species present but with the addition of K^+^ cations, there is a competition effect at the double layer which may impact the degree of alignment. This is consistent with the effect reported by Lee et al., who selectively tailored the double layer in monoethanolamine containing electrolytes by introducing alkali cations to enable the direct electrochemical conversion of the amine–CO_2_ adduct.^14^

The effect of CO_2_ capture by the PEI on the mechanical surface properties was also explored. The CO_2_ capture mechanism of amine solutions has been reported in the literature^67−69^ to occur via the reactions below, where a bond is formed based on nucleophilic attack on CO_2_ carbon by an amine (Lewis base).

For primary (R_1_NH_2_) and secondary amines (R_1_R_2_NH), the overall reaction is given by eq 3. The mechanism is a two-step reaction, which involves first the formation of a zwitterion intermediate, followed by transfer of an amine proton to a free base resulting in carbamate formation.
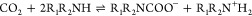
3

For tertiary amines (R_1_R_2_R_3_N), the overall reaction mechanism can be represented by the eq 4. In this case, the zwitterion can only be deprotonated by water molecules since tertiary amines do not have a free proton. Thus, the tertiary amine deprotonates water, which then reacts with CO_2_ forming ammonium bicarbonate.
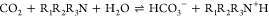
4

The PEI in this study contains primary, secondary, and tertiary amines at an approximate ratio of 40/36/24. Thus, carbamate formation dominates the CO_2_ capture mechanism in these materials.^30^

**Figure 5:**
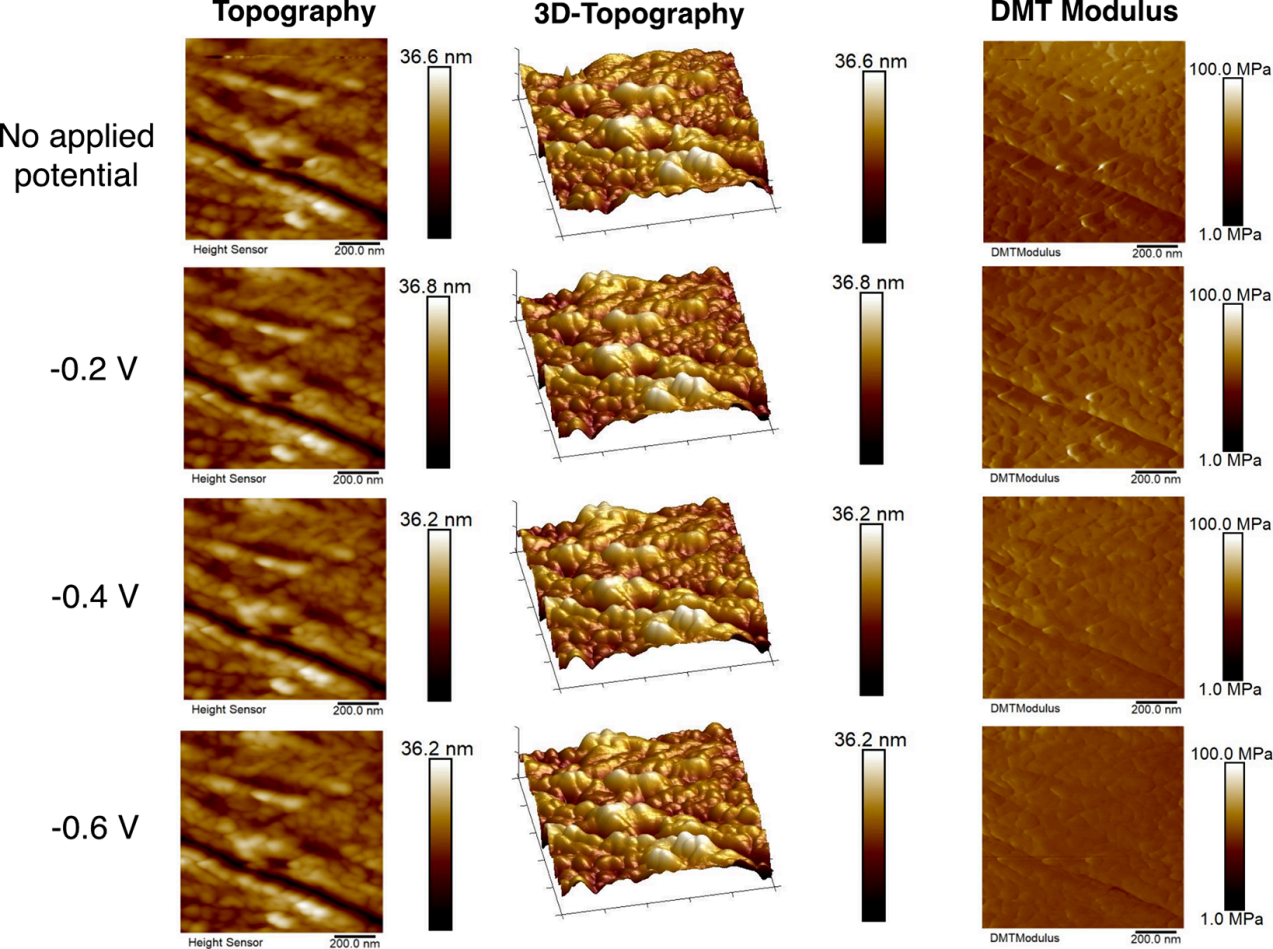
Topography and mechanical property mapping of Ag surface with PEI addition + 0.1 *m* KHCO_3_ (CO_2_ saturated) under no applied potential and with applied potentials in the range of −0.2 V to −0.6 V vs RHE. All of the images have a scan area of 1 × 1 μm^2^. Scale bars are shown at the right side of each image. All voltages quoted vs RHE.

Figure 5 shows the topographical and mechanical property maps of the CO_2_ loaded electrolytes. The average values of the surface roughness, DMT modulus, and surface deformation are given in Table S3. In this case, the DMT modulus exhibited a moderate increase under negative applied potentials, although again it was less significant than in the case of the PEI electrolyte with no supporting electrolyte salt or CO_2_ saturation, and remained relatively constant as increasingly negative potentials were applied. This suggests less of an alignment of the polymer chains with the electrode surface in this case, likely due the polymeric chains now containing the carbamate anion. The effect of this negatively charged species along the polymeric backbone, along with the presence of the supporting electrolyte previously described, may decrease the ability of the polymer chains to collapse, resulting in the average DMT surface modulus remaining more constant.

**Figure 6:**
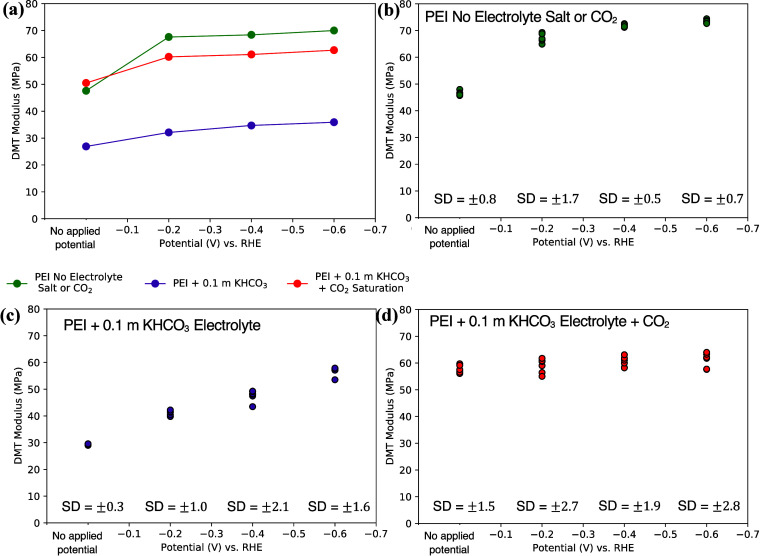
Summary of effect of composition on changes in modulus of PEI MW 2000 electrolyte. (a) Modulus evolution of PEI MW 2000 electrolyte in the potential range studied. (b–d) DMT modulus values for 6 spots on AFM image their standard deviations (SD) for (b) PEI MW 2000 electrolyte with no electrolyte salt or CO_2_, (c) PEI MW 2000 + 0.1 *m* KHCO_3_ electrolyte, and (d) PEI MW 2000 + 0.1 *m* KHCO_3_ + CO_2_ saturation electrolyte in the potential range studied.

Figure 6a summarizes the evolution of the average DMT modulus under applied potential for the PEI MW 2000 electrolyte in each of the three electrolyte conditions studied. Figure 6b–d shows the DMT modulus values for each of the 6 spots taken over one of the 1 × 1 μm^2^ images captured for each electrolyte case, along with the calculated standard deviations (SD), to provide an indication of the error in the data reported. The DMT values were found to be uniform across the sample surfaces analyzed.

**Figure 7:**
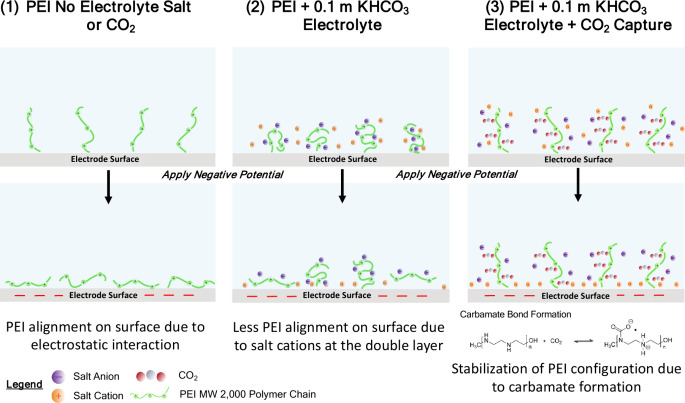
Schematic representation of hypothesized changes in PEI MW 2000 configuration at the electrode surface at different electrolyte compositions.

Figure 7 is a schematic summary of the proposed changes in PEI conformation at the Ag surface under negative applied potentials based on the trends in nanomechanical properties described. In case 1, PEI electrolyte with no supporting electrolyte ions or CO_2_ capture, the increase in the “alignment effect” of the cationic PEI chains on the electrode surface due to the electrostatic interaction was the most pronounced, with no competition with other cationic species at the electrical double layer. This translates to an increase of the average DMT modulus of the surface. With the addition of supporting electrolyte salt ions (case 2), the observed increase in the modulus was diminished, suggesting a competition between cations and PEI chains at the negatively charged surface. With the introduction of CO_2_ capture in the system (case 3), the modulus change was also less pronounced under applied potentials, indicating that the relative “stabilization” of the PEI charges via carbamate bond formation also impacts the ability of the polymeric chains to change their structural conformation at a charged interface. These significant structural differences of PEI at the electrode surface under applied potential show that the transport and reaction behaviors at the electrolyte and electrode are complex and require careful in situ or operando measurement and modeling to optimize the electrolyte system for combined CO_2_ capture and conversion.

### 3.4 Effect of Polymer Molecular Weight

**Figure 8:**
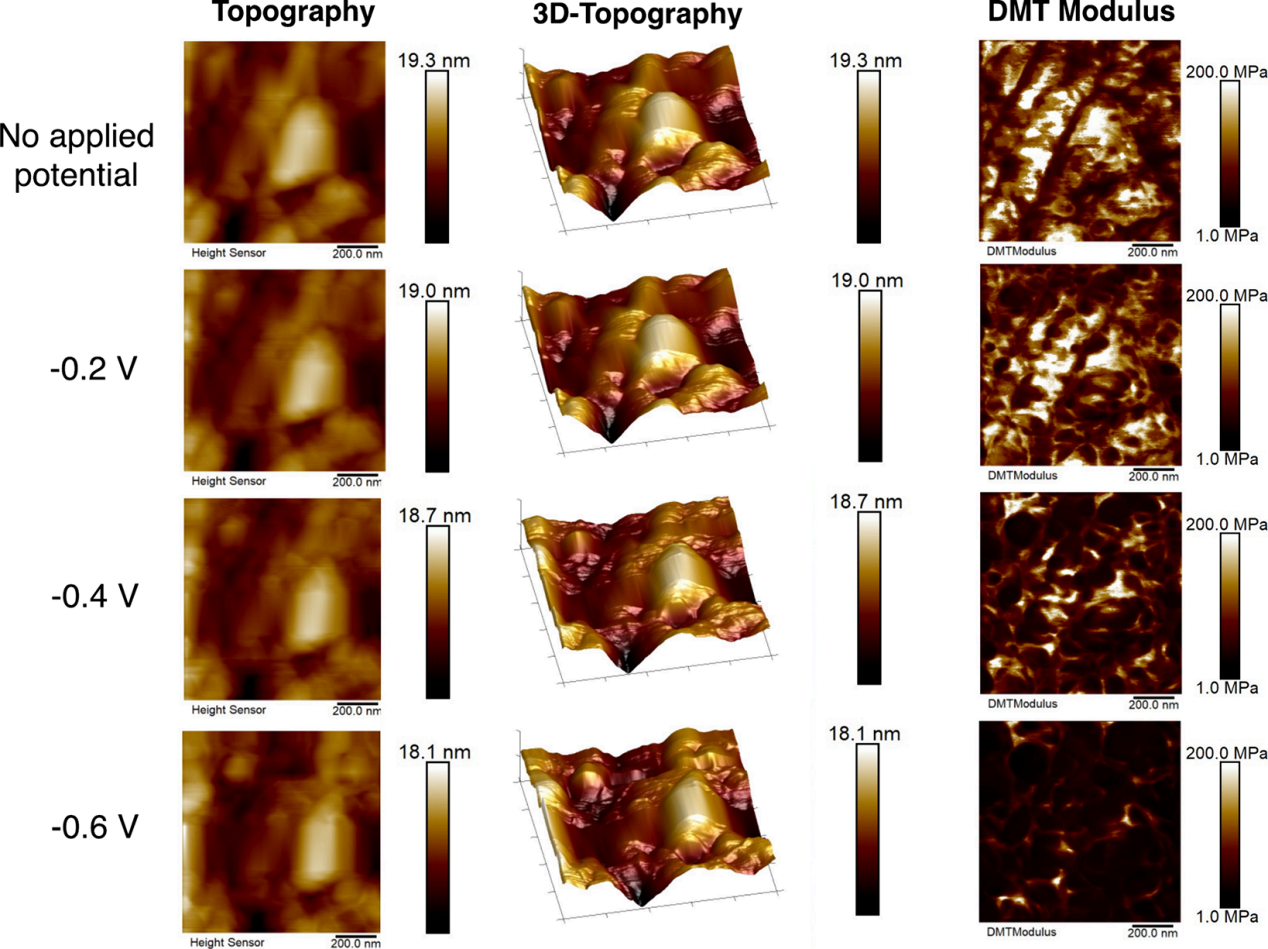
Topography and mechanical property mapping of Ag surface with PEI MW 25000 addition under no applied potential and with applied potentials in the range of −0.2 V to −0.6 V vs RHE. All of the images have a scan area of 1 × 1 μm^2^. Scale bars are shown at the right side of each image. All voltages quoted vs RHE.

Since the MW of PEI studied in the earlier sections is only 2000, these molecules are short brushes, and there are limited structural conformations that they can exhibit. Thus, we studied PEI with significantly larger MW to investigate possible effects of greater inter- and intramolecular interactions of PEI under applied potential. The effect of varying polymer MW on the changes in polymer conformation at the surface was investigated by conducting the same experiments outlined with PEI MW 25000. The two samples (PEI MW 2000 and PEI MW 25000) were normalized by number of polymer chains in solution. Figure 8 shows the topography and nanomechanical properties of the PEI MW 25000 electrolyte with no applied potential, as well as under increasingly negative applied potentials of −0.2 V, −0.4 V, and −0.6 V vs RHE. The average values of the surface roughness, DMT modulus, and surface deformation are given in Table S4. A few distinct features were notable compared to the case of PEI MW 2000. First, the average DMT modulus of the surface without applied potential was higher in this case (>100 MPa, compared to ∼50 MPa). This is possibly attributed to the longer, more rigid polymeric backbone compared to the shorter MW 2000 polymer brushes. Under applied potential, the trend in the change of the average DMT is the opposite as in the case of PEI MW 2000; while we previously found an *increase* in surface modulus as a negative potential was applied, here we observed a *decrease*. This may be attributed to the increased thickness of the adsorbed layer relative to the case of the low MW polymer. Since the PEI MW 25000 chains are much longer and more flexible relative to the PEI MW 2000, polymer “blobs” may form in this case, a phenomenon that has been reported for higher MW polymers in the literature.^70^ As the chains collapse on the electrode surface due to the electrostatic interaction, dangling loops and tails may form extending away from the electrode surface, and multiple layers of entangled polymers may be present. This is in contrast to the less flexible and shorter PEI chains that can fully collapse on the Ag and could explain the surface becoming “softer” as the electrode is polarized, resulting in a decrease in the average DMT modulus. These results may help understand the impact of catalyst/polymer interactions at concentrations which may replicate electrodes with higher ionomer to catalyst ratios used in electrolyzers.

**Figure 9:**
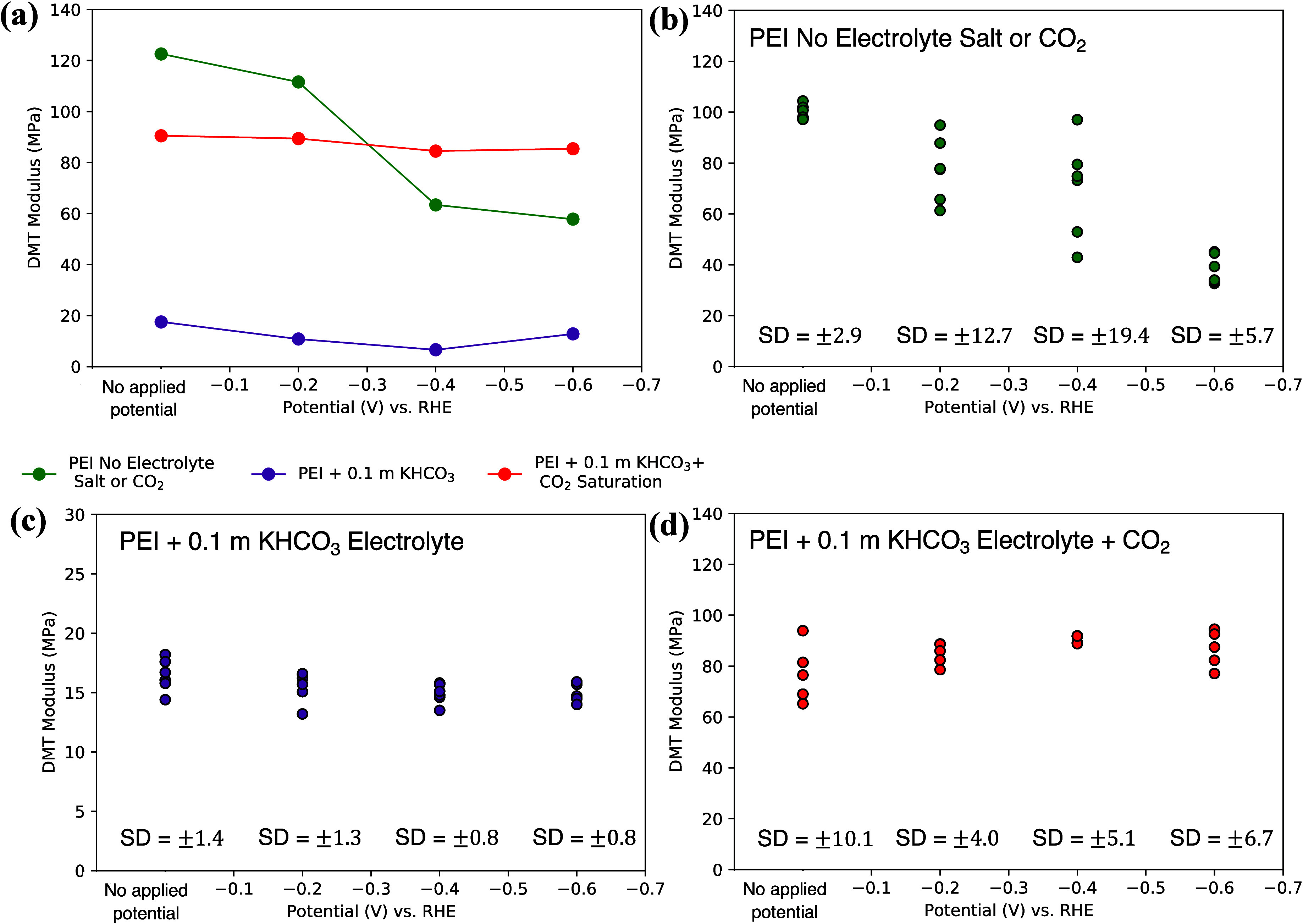
Summary of effect of composition on changes in modulus and configuration of PEI MW 25000 electrolyte. (a) Modulus evolution of PEI MW 25000 electrolyte in the potential range studied. (b–d) DMT modulus values for 6 spots on AFM image and standard deviations (SD) for (b) PEI MW 25000 electrolyte with no electrolyte salt or CO_2_, (c) PEI MW 25000 + 0.1 M KHCO_3_ electrolyte and (d) PEI MW 25000 + 0.1 M KHCO_3_ + CO_2_ saturation electrolyte in the potential range studied.

As with the case of the PEI MW 2000, the effect of supporting electrolyte salt and CO_2_ capture was also explored. The topography and mechanical property mapping for the electrolyte with the addition of supporting electrolyte salt and CO_2_ saturation are shown in Figure S1 and Figure S2. The average values of the surface roughness, DMT modulus, and surface deformation are given in Table S5 and Table S6. Figure 9a summarizes the evolution of the average DMT modulus under applied potential for the PEI MW 25000 electrolyte in each of the three electrolyte conditions studied. Figure 9b–d shows the DMT modulus values for each of the 6 spots taken over one of the 1 × 1 μm^2^ images captured for each electrolyte case, along with the calculated standard deviations (SD), to provide an indication of the error in the data reported. In the case of PEI MW 25000 with 0.1 *m* KHCO_3_ salt addition, the change of the average DMT modulus was minimized, as occurred in the case of PEI MW 2000. The average DMT modulus of the surface was significantly lower than that of the surface without salt, which as explained previously, is attributed to the salt ions screening some of the charges on the polymeric backbone leading to a more clumped polymer conformation. We again observed a small decrease in DMT modulus with applied potential (attributed to the effect described above without salt), but the change was less pronounced than in the case of no supporting electrolyte salt, possibly due to the presence of salt ions at the electrode surface. In the case of electrolyte salt addition and CO_2_ capture, the effect was similar to that of the lower MW polymer, with the DMT modulus remaining relatively constant with applied potential. This may be due to carbamate bond formation. In this case, the standard deviations, particularly in the case of PEI MW 25000 with no electrolyte additives, were significantly higher overall than in the case of the PEI MW 2000. This suggests less overall uniformity of interfacial properties with the addition of the longer polymeric chains in the electrolyte, consistent with the possible formation of dangling loops and tails described above.

**Figure 10:**
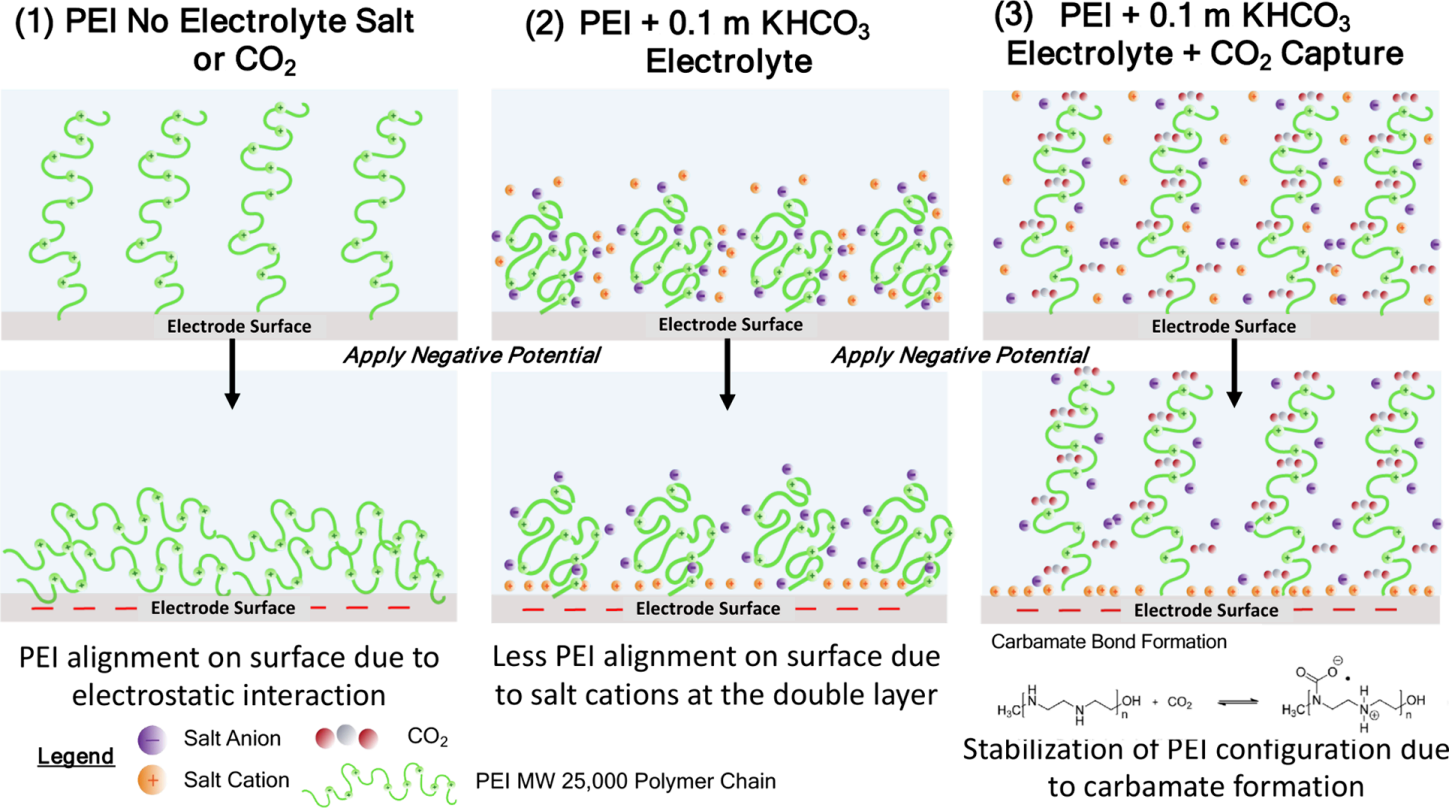
Schematic representation of hypothesized changes in PEI MW 25000 configuration at the electrode surface at different electrolyte compositions.

The phenomena described are summarized in Figure 10. From these results, it is evident that polymer molecular weight impacts the changes in conformation occurring at the surface, which results in different trends in the average DMT modulus detected by AFM. As in the case of the PEI MW 2000 polymer, the electrolyte composition (presence of supporting electrolyte salt and CO_2_ capture) impacts the changes in surface modulus detected.

## 4 Conclusions

This study revealed that the addition of an amine-based polymer (PEI) for tandem CO_2_ capture and conversion impacts the near-electrode environment on a Ag planar electrode during CO_2_R. The change in surface morphology of the Ag electrode with PEI addition was studied in situ using EC-AFM. Surfaces containing the PEI electrolyte additive could clearly be distinguished from Ag surfaces based on their differences in mechanical properties (orders of magnitude differences in the DMT modulus). Applying a negative polarization on the Ag electrode surface resulted in changes in the DMT surface modulus, suggesting reorganization of the PEI chains on the electrode surface. The electrostatic interaction between the positively charged PEI chains and the negatively charged surface leads to a realignment of the polymer chains, where they become oriented parallel to the electrode surface, increasing the average modulus of the surface. This reorganization effect is dependent on the electrolyte composition: the addition of supporting electrolyte salt reduces the extent of alignment of PEI on the electrode surface, possibly due to a competition of the salt ions for charged sites. The effect is also modulated by the formation of carbamate bonds upon CO_2_ capture by the electrolyte solution, suggesting that the PEI is more rigid in this case and exhibits less of an alignment on the electrode surface. The effect of polymer molecular weight was also explored, and it was found that PEI of MW 25000 showed a different behavior on the charged surface under polarization. Under applied potential in this case, the DMT modulus of the surface decreased. This is attributed to the longer and more flexible polymer chains forming a denser adsorbed layer at the surface, with loops and tails forming and multiple possible adsorption layers, as the polymer approaches the surface. This is the first study of its kind to probe the behavior of PEI at an electrocatalyst surface using in situ EC-AFM and provides information exclusively about the morphology of this interfacial region. Future work is required to establish a more detailed mechanism of the adsorption and CO_2_ conversion reaction pathways of these materials at charged electrode surfaces using tools such as in situ or operando spectroscopy (i.e., ATR-SEIRAS), which can provide more insights into the chemical nature of the interface. Overall, the findings from this study provide fundamental insights into the behavior of polymeric electrolytes being developed for combined CO_2_ capture and electrochemical conversion. The polymer conformational changes elucidated in this work may impact electrochemical performance, and future studies are required to fully discern the structure–property relationships in PEI-based electrolytes. These findings highlight the complexity of interfacial phenomena in systems combining CO_2_ capture and electrochemical conversion that call for in situ and operando characterization to help guide the design of next-generation electrolyte materials for this process.

## Supporting Information Available

Root mean square surface roughness (*R*_q_) estimation, calculation of modulus by Derjaguin–Muller–Toporov (DMT) model, properties of Ag surface with PEI MW 2000 and MW 25000 addition under applied potentials, topography and mechanical property mapping of Ag surface with PEI MW 25000 with KHCO_3_ addition and CO_2_ saturation under applied potentials (PDF)
