# Supplementary material for: Electrolyte–Electrocatalyst Interfacial Effects of Polymeric Materials for Tandem CO2 Capture and Conversion Elucidated Using In Situ Electrochemical AFM
Source: ACS Appl Mater Interfaces. 2024 Aug 1;16(32):42021–33. doi: 10.1021/acsami.4c01908 (PMC11331441; doi:10.1021/acsami.4c01908)
Supplement: Supplementary file 1 — am4c01908_si_001.pdf [file am4c01908_si_001.pdf]

Supporting Information

Electrolyte-Electrocatalyst Interfacial Effects of Polymeric Materials for Tandem CO_2 Capture and Conversion Elucidated Using In Situ Electrochemical AFM

Sara T. Hamilton[1], Maria Kelly[2,3], Wilson A. Smith[2,3*] and Ah-Hyung Alissa Park[4*]

[1]Department of Earth and Environmental Engineering, Columbia University, New York, NY 10027, USA
[2]National Renewable Energy Laboratory, Golden, Colorado 80401, USA
[3]Department of Chemical and Biological Engineering and Renewable and Sustainable Energy Institute, University of Colorado Boulder, Boulder, Colorado 80309, USA
[4]Department of Chemical and Biomolecular Engineering, University of California Los Angeles, Los Angeles, California 90024, USA

Corresponding authors:
Wilson A. Smith: Wilson.Smith@nrel.gov
Ah-Hyung Alissa Park: apark@seas.ucla.edu

Root mean square surface roughness (R_q) estimation

The root mean square surface roughness (R_q) was estimated using Nanoscope software from each of the topography images (scan area 1 x 1 µm) obtained using the AFM. R_q represents the standard deviation of the distribution of surface heights and its mathematical definition is given by:

$$R_q = \sqrt{\frac{1}{L}\int_0^L |(y(x))^2|\, dx}$$

Where L is the length of the profile on the x-axis and y(x) is the variation of the height from the profile line for each data point.

Calculation of Modulus by Derjaguin-Muller-Toporov (DMT) Model

Force distance curves are obtained during imaging of sample surfaces using Peak Force QNM tapping mode, and the force curves can be converted to a force versus separation plot. The retract curve is fit using the Derjaguin-Muller-Topotov (DMT) model to obtain the reduced modulus, E^* :

$$F - F_{adh} = \frac{4}{3} E^* \sqrt{R(d-d_o)^3}$$

Here, $F - F_{adh}$ is the force of the cantilever relative to
the adhesion force, R is the radius of the tip, and $d - d_o$ is the deformation of the sample.

Table S1. Properties of Ag surface with 8 wt.% PEI MW 2,000 addition under no applied potential and with applied potential of -0.2V, -0.4 V and -0.6 V vs. RHE.

Potential applied vs. RHE	Average Roughness (R_q), nm	Average Modulus, MPa	Average Deformation, nm
No applied potential	5.9 ± 2.7	47.6 ± 2.1	8.6 ± 1.3
-0.2 V	5.4 ± 3.6	67.6 ± 8.7	8.3 ± 0.3
-0.4 V	6.6 ± 5.1	68.4 ± 5.2	7.7 ± 0.7
-0.6 V	6.6 ± 5.2	70.0 ± 2.6	7.9 ± 0.2

Table S2. Properties of Ag surface with 8 wt.% PEI MW 2,000 + 0.1 m $KHCO_3$ addition under no applied potential and with applied potential of -0.2V, -0.4 V and -0.6 V vs. RHE.

Potential applied vs. RHE	Average Roughness (R_q), nm	Average Modulus, MPa	Average Deformation, nm
No applied potential	6.8 ± 3.2	26.9 ± 2.7	11.0 ± 1.8
-0.2 V	5.2 ± 4.6	32.1 ± 7.7	10.6 ± 2.3
-0.4 V	6.3 ± 3.6	34.7 ± 11.5	10.7 ± 1.8
-0.6 V	6.5 ± 4.0	35.9 ± 18.3	11.0 ± 1.5

Table S3. Properties of Ag surface with 8 wt.% PEI MW 2,000 + 0.1 m $KHCO_3$ addition (CO_2 saturated) under no applied potential and with applied potential of -0.2V, -0.4 V and -0.6 V vs. RHE.

Potential applied vs. RHE	Average Roughness (R_q), nm	Average Modulus, MPa	Average Deformation, nm
No applied potential	5.7 ± 1.4	50.5 ± 9.9	8.5 ± 0.2
-0.2 V	6.2 ± 2.2	60.2 ± 7.0	6.7 ± 3.3
-0.4 V	6.2 ± 2.3	61.1 ± 7.5	7.4 ± 3.4
-0.6 V	6.8 ± 3.3	62.7 ± 11.9	7.4 ± 2.5

Table S4. Properties of Ag surface with 8 wt.% PEI MW 25,000 MW addition under no applied potential and with applied potential of -0.2V, -0.4 V and -0.6 V vs. RHE.

Potential applied vs. RHE	Average Roughness (R_q), nm	Average Modulus, MPa	Average Deformation, nm
No applied potential	5.5 ± 4.0	122.6 ± 36.0	7.9 ± 3.2
-0.2 V	5.3 ± 4.0	111.6 ± 46.6	7.8 ± 4.1
-0.4 V	5.0 ± 3.9	63.4 ± 38.5	8.2 ± 2.7
-0.6 V	6.1 ± 4.5	57.8 ± 4.6	9.0 ± 2.6

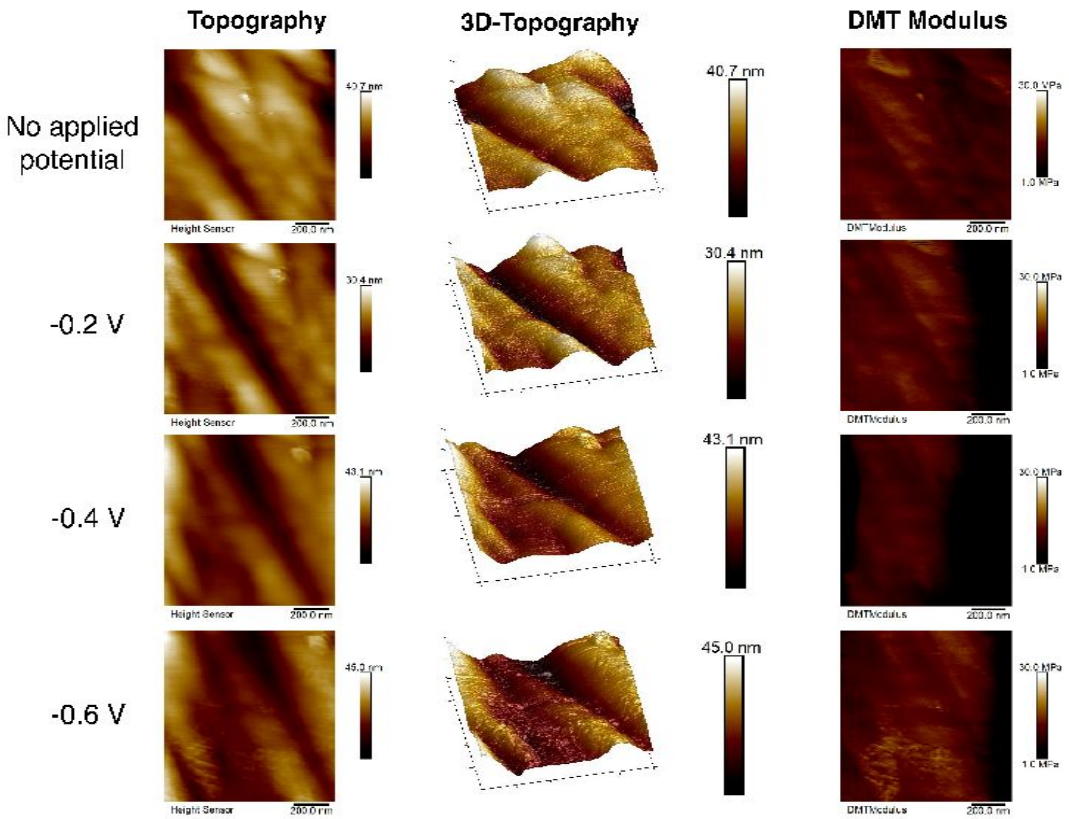

Figure S1. Topography and mechanical property mapping of Ag surface with PEI MW 25,000 + 0.1 M $KHCO_3$ addition under no applied potential and with applied potentials in the range of -0.2 V to -0.6 V vs. RHE. . All of the images have a scan area of 1 × 1 μm^2. Scale bars are shown at the right side of each image. All voltages quoted vs RHE.

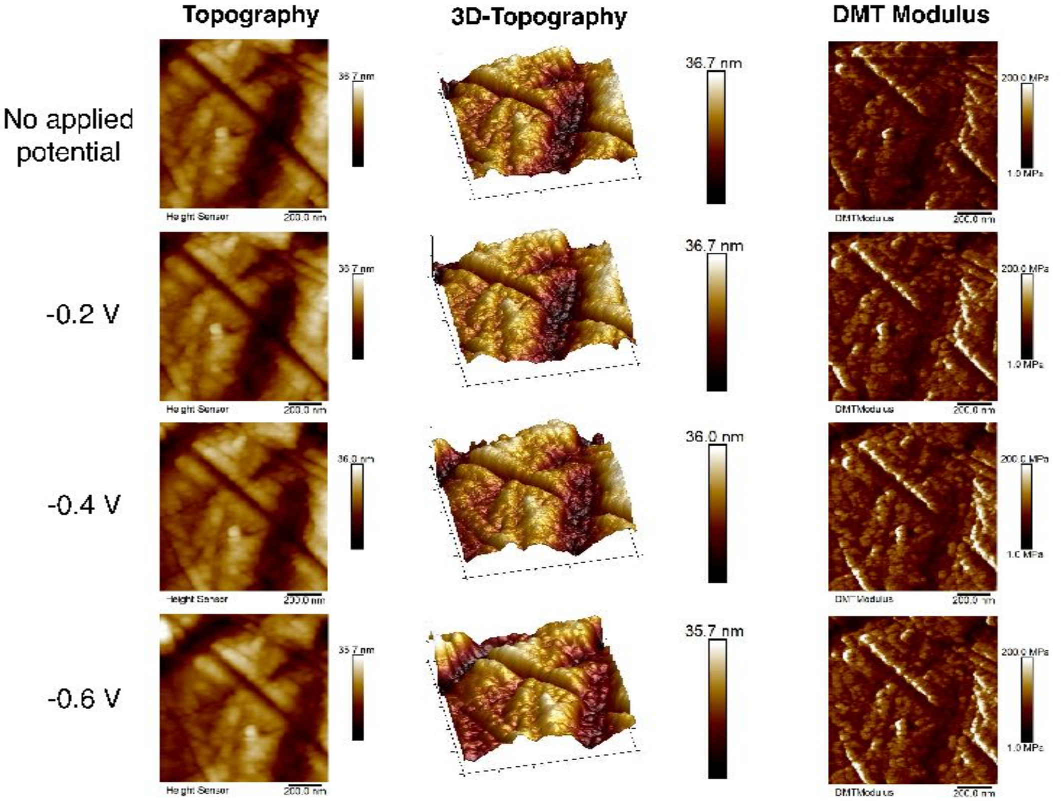

Figure S2. Topography and mechanical property mapping of Ag surface with PEI MW 25,000 addition + 0.1 M $KHCO_3$ (CO_2 saturated) under no applied potential and with applied potentials in the range of -0.2 V to -0.6 V vs. RHE. . All of the images have a scan area of 1 × 1 μm^2. Scale bars are shown at the right side of each image. All voltages quoted vs RHE.

Table S5. Properties of Ag surface with 8 wt.% PEI MW 25,000 + 0.1 m $KHCO_3$ addition under no applied potential and with applied potential of -0.2V, -0.4 V and -0.6 V vs. RHE.

Potential applied vs. RHE	Average Roughness (R_q), nm	Average Modulus, MPa	Average Deformation, nm
No applied potential	5.8 ± 0.8	17.5 ± 7.6	19.0 ± 14.2
-0.2 V	6.1 ± 2.1	10.8 ± 4.2	40.9 ± 9.9
-0.4 V	6.8 ± 3.3	6.6 ± 4.8	42.1 ± 25.2
-0.6 V	7.2 ± 3.2	12.8 ± 1.9	21.3 ± 11.1

Table S6. Properties of Ag surface with 8 wt.% PEI MW 25,000 + 0.1 m $KHCO_3$ addition (CO_2 saturated) under no applied potential and with applied potential of -0.2V, -0.4 V and -0.6 V vs. RHE.

Potential applied vs. RHE	Average Roughness (R_q), nm	Average Modulus, MPa	Average Deformation, nm
No applied potential	5.5 ± 1.8	90.5 ± 11.2	6.7 ± 1.1
-0.2 V	5.7 ± 1.5	89.4 ± 16.5	7.0 ± 0.3
-0.4 V	6.0 ± 2.1	84.2 ± 11.0	6.9 ± 0.8
-0.6 V	6.6 ± 3.3	85.4 ± 24.7	7.1 ± 0.9
